# BLV-CoCoMo-qPCR: comparison of other detection methods for BLV infection and kinetics analysis in experimental transmission of BLV in cattle

**DOI:** 10.1186/1742-4690-8-S1-A21

**Published:** 2011-06-06

**Authors:** Mayuko Jimba, Shin-nosuke Takeshima, Yuki Matsumoto, Naohiko Kobayashi, Tamako Matsuhashi, Junko Kohara, Yoko Aida

**Affiliations:** 1 Viral Infectious Diseases Unit, RIKEN, Wako, Saitama 351-0198, Japan; 2 Laboratory of Viral Infectious Diseases, Department of Medical Genome Sciences, Graduate School of Frontier Science, The University of Tokyo, Wako, Saitama 351-0198, Japan; 3 Gifu Prefectural Livestock Research Institute, Takayama, Gifu 506-0101, Japan; 4 Hokkaido Animal Research Center, kamikawa-gun, Hokkaido, 080-0038, Japan

## 

Bovine leukemia virus (BLV) infects cattle worldwide, imposing a severe economic impact on the dairy cattle industry. BLV is the etiological agent of enzootic bovine leukosis. Recently we developed a new quantitative real-time PCR method using Coordination of Common Motifs (CoCoMo) primers to measure the proviral load of known and novel BLV variants in clinical animals [1]. In this study, we analyzed a kinetic of the provirus and relevance of the BLV antibody titer.

First, we experimentally compared the sensitivity of our methods with previously reported for BLV provirus detection real-time PCR system, and determined the high sensitivity of our developed BLV-CoCoMo-qPCR.

Next, we estimated the sensitivities of the antibody detection methods such as ELISA, PHA and AGID and the provirus load estimated by BLV-CoCoMo-qPCR, using a total of 391 cattle. In three methods, high false-negative rate were observed at the range (100 copies/10^5^cells) of low provirus copy number cattle. Meanwhile, a number of cattle with high antibody titer cannot be detected provirus. To investigate the reasons for the results, two cattle were experimentally infected with BLV and followed-up the titer of serum antibody and proviral load. We detected that proviral load were suppressed at the high antibody titer stage and it increased at the low antibody titer stage.

In this study, we clearly detected the inverse proportion between antibody titer and proviral load for the first time in natural host.

It suggested that quantification of proviral load is very important to halt the spread of BLV.
